# A Comparison of Tissue versus Swab Culturing of Infected Diabetic Foot Wounds

**DOI:** 10.1155/2016/8198714

**Published:** 2016-03-30

**Authors:** Ying Huang, Ying Cao, Mengchen Zou, Xiangrong Luo, Ya Jiang, Yaoming Xue, Fang Gao

**Affiliations:** Department of Endocrinology and Metabolism, Nanfang Hospital, Southern Medical University, Guangzhou 510515, China

## Abstract

*Objective*. To compare the efficacy of swabbing versus tissue biopsy for microbiological diagnosis of diabetic foot infection.* Methods*. This was a prospective trial. Fifty-six patients with diabetic foot infection were divided into the following 3 groups according to the PEDIS grading system: grade 2 (*n* = 10), grade 3 (*n* = 29), and grade 4 (*n* = 17). Two specimens were collected from each wound for microbial culturing after debridement, including a superficial swab and a deep tissue punch biopsy specimen.* Results*. Swab culturing identified all of the microorganisms isolated from the corresponding deep tissue specimens in 9/10 of grade 2 wounds (90.0%), and this proportion decreased to 12/29 (41.4%) and 7/17 (41.2%) for grades 3 and 4 wounds, respectively (*p* = 0.02). Moreover, the sensitivity for identifying Gram-negative bacteria, such as* E. coli *and* Citrobacter*, by swabbing was low (33.3%). In addition, some Gram-negative bacteria, such as* Serratia* and* Ralstonia pickettii*, were isolated from deep tissues but not from swabs.* Conclusions*. Swab culturing may be reliable for identification of pathogens in diabetic foot wounds classified as grade 2. However, it is advisable to culture deep tissue specimens for wounds of grade ≥3 because swab culturing is associated with a high risk of missing pathogens, especially Gram-negative bacteria.

## 1. Introduction

Diabetes patients have a 12–25% risk of developing a foot ulcer during their lifetime [1, 2]. Infection is a frequent (40–80%) and costly complication of diabetic foot ulcer and represents a major cause of morbidity and mortality [3]. Consequently, causative organisms need to be reliably diagnosed and promptly controlled.

The techniques used for sampling and microbiological analysis strongly affect the quality of evaluations of the microbiota in diabetic foot wounds. The Infectious Diseases Society of America recommends that an appropriate specimen for culturing should be obtained from diabetic foot infections (DFIs) to guide antibiotic therapy [4]. For identification of pathogens, the International Working Group on Diabetic Foot (IWGDF) has proposed that clinicians should obtain cultures from tissue specimens rather than from swabs [5, 6]. Swabs are often contaminated with normal skin flora or colonizers, and their use may result in failure to identify deep tissue pathogens [7]. However, superficial wound swabbing is more widely applied in clinical practice because it is easy to perform and noninvasive. We questioned whether there is any difference in the microbiological results obtained between the two sampling techniques. A comparison of culture results from swab and tissue specimens among wounds with infections of varying severity has not yet been performed. Furthermore, the diagnostic accuracy of swab culturing in identifying different types of microorganisms has not been previously examined.

With the aim of reappraising the reliability of superficial swabbing for the culturing of diabetic foot wounds with infections of varying severity, we prospectively compared the following two microbiological sampling techniques for the routine identification of pathogens: superficial swabbing and deep tissue biopsy.

## 2. Patients and Methods

### 2.1. Patients

We enrolled 56 consecutive diabetic patients with clinically infected foot ulcers. The patients were hospitalized at the Department of Endocrinology and Metabolism of Nanfang Hospital affiliated with Southern Medical University from October 2014 to July 2015. The study was approved by the ethics committee of Nanfang Hospital, and informed consent was obtained from all patients.

Clinical diagnosis of infection was defined by the presence of at least 2 of the following indicators: local swelling or induration, >0.5 cm of erythema around the wound, local tenderness or pain, local warmth, and purulent discharge [4, 5, 8]. Clinical severity of infection was quantified according to the infection category of the PEDIS system proposed by the IWGDF [5] as follows: grade 1 wounds were uninfected; grade 2 wounds were mildly infected, involving only the skin or subcutaneous tissue, as well as any erythema present extended <2 cm; grade 3 lesions were moderately infected, involving structures deeper than the skin and subcutaneous tissues (e.g., bone, joint, tendon, and muscle) or erythema extending >2 cm from the wound margin; and grade 4 wounds were severely infected, including any foot infection with systemic inflammatory response syndrome. The patients were classified into 3 groups based on this system, including 10, 29, and 17 patients with grades 2, 3, and 4 wounds, respectively.

### 2.2. Specimen Collection and Microbiological Culturing

Two specimens were collected from each wound after the wound had been cleansed (using sterile saline and gauze) and debrided (removal of necrotic tissue, foreign material, calluses, and undermined wound edges) [9]. No antimicrobial agent (e.g., alcohol or iodine) or antiseptic was introduced into the wound before specimen collection. Each wound was swabbed using the Levine technique, involving rotation of a wound swab over a 1 cm^2^ area of the wound for 5 seconds, using sufficient pressure to extract fluid from the inner part of the wound [10–12]. A deep tissue specimen of 4 mm in diameter was obtained from the base of the ulcer via punch biopsy [13, 14]. The specimens were placed into sterile transport containers and sent to the microbiology laboratory for aerobic culturing within 20 minutes. Anaerobic culturing was not performed in this study. Cultures were processed following the same standard procedures for the swab and tissue samples [15, 16]. Most of the bacterial isolates were identified using a BD Phoenix system, and a few isolates were identified manually.* Candida* isolates were identified using color display plates. All of the culture results for the swab and tissue specimens were reported in the same manner, with specification of each isolate.

### 2.3. Statistical Analysis

Statistical analysis was performed with Statistical Package for Social Sciences version 19 (IBM SPSS, Chicago, IL). The distribution of data was evaluated for normality using the Kolmogorov-Smirnov test. Normally distributed variables were expressed as the mean ± standard deviation and compared by one-way ANOVA. Variables without normal distribution were expressed as the median (interquartile range) and compared by Kruskal-Wallis H test. Qualitative variables were compared using *χ* ^2^ test. Variation trends of variables among three groups were evaluated by linear regression (for quantitative variables with normally distribution), Spearman correlation test (for quantitative variables without normally distribution), and linear-by-linear association (for qualitative variables). Statistically significant differences were indicated by a *p* value of <0.05.

## 3. Results

### 3.1. Characteristics of Patients and Wounds

Compared with the patients with grade 2-3 diabetic foot wound, those with a grade 4 lesion had significantly decreased albumin (*p* = 0.028) and a higher white blood cell count (*p* < 0.001), neutrophil count (*p* < 0.001), and CRP level (*p* < 0.001). With an increasing PEDIS grade, the proportion of patients with renal impairment had an increased trend (*p* = 0.019), but the difference was not significant statistically (*p* = 0.059). A total of 33.9% of the enrolled patients had recently received antimicrobial therapy (within the preceding 7 days) at the time of specimen collection. There were no significant differences in the majority of the clinical characteristics examined (gender, age, duration of diabetic foot ulcers, previous antibiotic use, etc.) among the groups with varying infection severity (Table 1).

### 3.2. Number of Pathogens Isolated

A total of 81 microorganisms (an average of 1.4 per wound) were isolated from both the swab and tissue specimens from 56 wounds. The prevalence of polymicrobial infection diagnosed by tissue culture increased from 20.0% for grade 2 wounds to 41.4% and 70.6% for grade 3 and grade 4 wounds, respectively (*p* = 0.029). There was little variation in the diagnosis of monomicrobial versus polymicrobial infection, using the two specimen collection techniques for the DFIs of different grades. Gram-positive bacteria were predominant in grades 2-3 wounds, as determined by either swab or tissue culturing. Separate analysis of grade 4 wounds comparing swab and tissue culturing demonstrated that Gram-positive bacteria were the most frequently isolated (57.1%) in the swab specimens, while Gram-negative bacteria were predominant in the deep tissue specimens (61.3%), but the difference was not significant statistically (*p* = 0.157) (Table 2).

### 3.3. Concordance between Swab and Tissue Cultures

**Figure 1:**
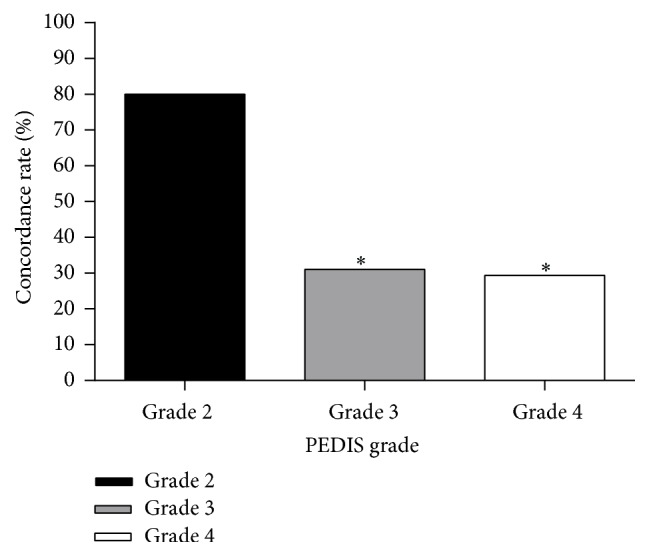
Concordance between culture results from swab and tissue specimens. ^*∗*^ *p* < 0.05 versus grade 2.

For grade 2 wounds, swabbing allowed for identification of all of the microorganisms isolated from the corresponding deep tissue specimens in 9/10 wounds (90.0%), whereas this proportion decreased to 12/29 (41.4%) and to 7/17 (41.2%) in grades 3 and 4 wounds, respectively (*p* = 0.02). The organisms isolated by ulcer swabbing and tissue biopsy were identical in 8/10 (80.0%) of grade 2 wounds, 9/29 of grade 3 wounds (31.0%), and 5/17 of grade 4 wounds (29.4%) (*p* = 0.014). The proportion of swab specimens lacking microorganisms isolated from the deep tissue specimens increased from 1/10 (10.0%) in grade 2 wounds to 17/29 (58.6%) and 10/17 (58.8%) in grades 3 and 4 wounds, respectively (*p* = 0.02) (Figure 1).

### 3.4. Microbial Load and Diagnostic Accuracy of Swab Cultures

*Staphylococcus aureus* was the most commonly isolated species, appearing in 26.8% of the swab specimens and in 28.6% of the tissue specimens. Among the Gram-negative organisms,* Proteus* spp. were the most prevalent, being isolated from 14.3% of the swabbed wounds and 17.9% of the biopsied wounds. Compared with the culture results for the tissue samples, the sensitivity and specificity for the identification of all types of Gram-positive bacteria by swab culturing were over 60% and 80%, respectively. Among the Gram-negative organisms, the sensitivity for the identification of* E. coli*,* Morganella*, and* Citrobacter* by swab culturing was very low (33.3%). Some pathogens isolated from the deep tissue specimens (such as* Serratia*,* Acinetobacter*,* Ralstonia pickettii*, and* Kluyvera ascorbata*) were not isolated from the swabs (Table 3).

## 4. Discussion

A reliable sampling technique is required to identify pathogens present in infected diabetic foot wounds. A systematic review of diagnosis of infections in diabetic foot ulcers has concluded that the available evidence is too weak to determine the optimal sampling technique [17]. To date, most researchers consider that tissue biopsy is the best method for the identification of pathogens in DFIs because deep biopsy is not prone to superficial contamination [4–6, 18, 19]. Another study has indicated that there is no need for biopsy, as there are no significant differences in the bacterial species isolated between swab and tissue samples [13]. Nelson et al. have carried out a large, prospective, multicenter trial to assess the concordance between culture results for swab and tissue specimens in patients with clinical DFIs, and the results of this completed trial will soon be published [9]. Previous studies have ignored the fact that the microbial species detected in wounds of varying depths and severities can significantly differ. Furthermore, the accuracy of swabbing has not been assessed with respect to the PEDIS infection grade. Thus, we reappraised the concordance between swab and tissue culturing according to the PEDIS infection grade of diabetic foot wounds.

We found that there was no significant difference in the mean number of isolates per specimen between swabbing and deep tissue biopsy of diabetic foot ulcers of different grades. Our results are consistent with those of some previous studies [13, 20–22]. However, in several other studies, the number of pathogens isolated by swabbing was significantly higher than those isolated by tissue biopsy or needle puncture [14, 23]. This discrepancy might be related to the cleansing or debridement step performed before collection of the specimens in our study. It might also be related to the Levine swabbing technique that we used. This technique involves rotation of the swab with sufficient pressure to extract fluid from the inner part of the wound. It thus may reduce superficial contamination and the omission of deep pathogens. In addition, with an increasing PEDIS grade, the prevalence of polymicrobial infection increased, while the dominant bacterial flora changed from Gram-positive bacteria to Gram-negative bacteria in tissue specimens. But the changes in the distributions of the bacterial strains according to the PEDIS grade differed between the swab and tissue cultures. It might be associated with debridement and antibiotic used before sampling, which might influenced culture results of swabs rather than that of tissues. van der Meer et al. have reported the differences in the repartition of the bacterial populations according to the Wagner grades [24]. However, the classification system used in that study was replaced by the PEDIS grading system in our study.

Our study revealed that the consistency of the microbiological results between the two sampling techniques decreased as the PEDIS infection grade increased. A total of 90.0% of the patients with grade 2 wounds would have been treated with antibiotics adequately based on the swab culture results alone. However, only 41.4% of those with grade 3 wounds and 41.2% of those with grade 4 wounds would have been adequately treated. In addition, the proportion of patients who may have been treated inadequately based on the swab culture results alone increased from 1/13 (10.0%) of those with grade 2 wounds to 19/31 (58.6%) of those with grade 3 wounds and 9/12 (58.8%) of those with grade 4 wounds. Hence, for grade 2 wounds, ulcer swabbing, which is easier to perform and relatively noninvasive, could be a satisfactory clinical sampling technique compared with deep tissue biopsy, which may carry a risk of injury to surrounding tissues, blood vessels, and nerves [13]. However, our data have demonstrated that it is necessary to perform tissue biopsy to obtain an accurate microbiological diagnosis of DFI to guide clinicians in choosing an appropriate antibiotic therapy for wounds of grade ≥3. Our results for wounds with infections of varying severity are in partial agreement with the results of Slater et al. [20], who have reported that 90% of swabs contained all of the organisms detected in tissue biopsies obtained from wounds not involving bone, but this value fell to 65% in cases with penetration of the wound into the bone or joint space. However, these authors excluded patients with infectious gangrene and did not assess the accuracy of swabbing with respect to the different PEDIS grades.

We found that* S. aureus* was the most commonly isolated species from both the swab and tissue cultures. These results are in agreement with previous findings [13, 20, 25–28]. Moreover, the sensitivity and specificity for the identification of all types of Gram-positive bacteria by swab culturing were over 60% and 80%, respectively, which are clinically acceptable values. However, the sensitivities for identification of Gram-negative bacteria such as* E. coli*,* Morganella*,* Acinetobacter*, and* Ralstonia pickettii* were unsatisfactory (0.0–33.3%). Gram-negative bacteria have been reported to be the predominant organisms in diabetic gangrene and deep wounds [22, 29, 30]. The low sensitivities for identification of these Gram-negative bacteria by swabbing observed in our study are probably attributable to the inability of swabbing to detect pathogens in deep lesions. In addition to cleansing, debridement or antibiotic use before sampling might alter the microbiology of specimens taken from the surface of a wound rather than from deep inside of the wound. Demetriou et al. [14] have reported 100.0% sensitivity and 14.3% specificity of swabbing for identification of pathogens in patients with neuropathic foot ulcer, in addition to 100.0% sensitivity and 18.2% specificity for that in patients with neuroischemic foot ulcer. The lower specificity of swab culturing demonstrated in these previous studies compared with ours is probably attributable to the presence of superficial contamination, whereas we collected each specimen after the wound had been thoroughly cleansed and debrided.

The major limitation of this study is the lack of anaerobic culturing compared with other investigations. Further study is required to evaluate the effectiveness of swab and tissue culturing in identifying anaerobes. In our study, the microbiological culture results for 3 patients with a clinically infected ulcer were negative. We speculate that these results are related to previous antibiotic use before specimen collection or to the absence of anaerobic cultures. A further limitation is the small number of included patients, especially those with grades 2 and 4 wounds. In addition, tissue specimens can more easily be obtained by curettage than by tissue biopsy. The curettage technique should have been used instead of tissue biopsy to obtain tissue specimens.

In conclusion, swab cultures may be reliable for guiding the antibiotic treatment of diabetic patients with grade 2 foot wounds. However, it is necessary to perform deep tissue biopsy for wounds of grade ≥3. In such cases, swab culturing is associated with a high risk of missing pathogens, especially Gram-negative bacteria.

## Figures and Tables

**Table 1:** Characteristics of patients with diabetic foot wounds of varying PEDIS grades.

Parameter	Grade 2	Grade 3	Grade 4	*p*
Number of patients	10	29	17	—
Type 1/type 2 DM	0/10	0/29	0/17	—
Sex (male/female)	5/5	18/11	12/5	0.565
Age (years)^a^	63.1 ± 11.6	60.4 ± 11.8	61.2 ± 11.8	0.824
Diabetes duration (years)^b^	10 (5.0–11.8)	10 (2.5–10.0)	10 (3.0–13.0)	0.956
Ulcer duration (days)^b^	30 (25–60)	30 (12–90)	30 (20–60)	0.956
Previous antibiotics use^c^	2 (20.0)	9 (31.0)	8 (47.1)	0.320
Retinopathy^c^	6 (60.0)	16 (55.2)	12 (70.6)	0.586
Renal impairment^c^	3 (30.0)	16 (55.2)	13 (76.5)	0.059
Neuropathy^c^	10 (100.0)	29 (100.0)	17 (100.0)	—
Lower limb arteriopathy^c^	2 (20.0)	10 (34.5)	7 (41.2)	0.531
Body mass index (kg/m^2^)^a^	24.9 ± 4.3	22.7 ± 2.3	25.1 ± 2.4	0.068
ABI^a^	1.0 ± 0.3	1.1 ± 0.3	0.9 ± 0.4	0.351
HbA_1c_ (%)^a^	9.8 ± 3.4	10.1 ± 2.7	9.4 ± 2.4	0.742
WBC (G/L)^a^	8.9 ± 2.2	8.1 ± 1.9	14.3 ± 4.2	0.000
Neutrophil (G/L)^a^	5.4 ± 1.7	5.6 ± 2.0	11.5 ± 4.0	0.000
CRP (mg/dl)^b^	7.2 (2.4–15.6)	21.3 (6.2–38.4)	62.0 (38.2–112.9)	0.000
Creatinine (mg/dL)^b^	84.5 (68.3–127.3)	83.0 (60.5–145.5)	88.0 (66.0–107.5)	0.912
LDL (mg/dL)^a^	2.8 ± 0.6	2.7 ± 0.8	2.4 ± 0.8	0.365
AST (IU/L)^a^	23.4 ± 8.1	19.4 ± 7.8	22.1 ± 15.9	0.54
ALT (IU/L)^a^	21.9 ± 9.9	17.8 ± 11.8	21.9 ± 16.6	0.501
Albumin (g/L)^a^	33.5 ± 4.3	30.9 ± 5.3	27.4 ± 7.0	0.028
FPG (mg/dL)^a^	6.6 ± 3.7	7.1 ± 3.4	8.1 ± 3.5	0.593

^a^Expressed as mean ± SD; ^b^expressed as median (1st quartile–3rd quartile); ^c^expressed as number (percentage). ABI, ankle-brachial index; LDL, low-density lipoprotein; ALT, alanine aminotransferase; AST, aspartate aminotransferase; CRP, C-reactive protein; FPG, fasting plasma glucose.

**Table 2:** Distribution of pathogens isolated from wounds of different PEDIS grade, *n* (%).

	Grade 2		Grade 3		Grade 4	
	Swab	Tissue	Swab	Tissue	Swab	Tissue
Number of pathogens species						
0	2 (20.0)	3 (30.0)	3 (10.3)	4 (13.8)	1 (5.9)	0 (0.0)
1	6 (60.0)	5 (50.0)	15 (51.7)	13 (44.8)	5 (29.4)	5 (29.4)
≥2	2 (20.0)	2 (20.0)	11 (38.0)	12 (41.4)	11 (64.7)	12 (70.6)
Classification of pathogens						
Gram-positive bacteria	8 (72.7)	8 (80.0)	26 (61.9)	23 (57.5)	16 (57.1)	10 (32.3)
Gram-negative bacteria	3 (27.3)	2 (20.0)	11 (26.2)	13 (32.5)	11 (39.3)	19 (61.3)
Fungi	0 (0.0)	0 (0.0)	5 (11.9)	4 (10.0)	1 (3.6)	2 (6.4)

**Table 3:** Microbial load and diagnostic utility of swab cultures.

Microorganism	Deep tissue (+)		Deep tissue (−)		Diagnostic utility of swab culture in identifying pathogens		
	Swab (+)	Swab (−)	Swab (+)	Swab (−)	SE (%)	SP (%)	Accuracy (%)
Presence of pathogens	46	4	3	3	92.0	50.0	87.5
Gram-positive organisms	29	3	5	19	90.6	79.2	85.7
*S. aureus*	12	4	3	37	75.0	92.5	87.5
*Enterococcus*	6	4	2	44	60.0	95.7	89.3
*Streptococci*	5	3	7	41	62.5	85.4	82.1
*CNS*	5	1	4	46	83.3	92.0	91.1
*Arcanobacterium pyogenes*	1	0	0	55	100.0	100.0	100.0
*Corynebacterium*	0	0	2	54	—	96.4	96.4
*Globicatella sanguis*	0	0	1	55	—	98.2	98.2
Gram-negative organisms	22	3	3	28	88.0	90.3	89.3
*Proteus*	7	4	1	44	63.6	97.8	91.1
*Pseudomonas*	3	0	2	51	100.0	96.2	96.4
*Klebsiella*	2	1	1	52	66.7	98.1	96.4
*Enterobacter*	2	0	2	52	100.0	96.3	96.4
*E. coli*	1	2	2	51	33.3	96.2	92.9
*Morganella*	1	2	0	53	33.3	100.0	96.4
*Citrobacter*	1	2	0	53	33.3	100.0	96.4
*Serratia*	0	2	0	54	0.0	100.0	96.4
*Acinetobacter*	0	1	0	55	0.0	100.0	98.2
*Myroides odoratimimus*	0	0	1	55	—	98.2	98.2
*Ralstonia pickettii*	0	1	0	55	0.0	100.0	98.2
*Kluyvera ascorbata*	0	1	0	55	0.0	100.0	98.2
Fungi	4	1	1	50	80.0	98.0	96.4

*S. aureus*: *Staphylococcus aureus*; *CNS*: *Coagulase-Negative Staphylococcus*.

SE: sensitivity; SP: specificity; (+): positive; (−): negative.
